# Extreme variability in the formation of chlorpyrifos oxon (CPO) in patients poisoned by chlorpyrifos (CPF)

**DOI:** 10.1016/j.bcp.2009.05.004

**Published:** 2009-09-01

**Authors:** Florian Eyer, Darren M. Roberts, Nicholas A. Buckley, Michael Eddleston, Horst Thiermann, Franz Worek, Peter Eyer

**Affiliations:** a Toxicological Department of the 2nd Medical Clinic, Technische Universität München, Ismaninger Str. 22, D-81664 Munich, Germany; b South Asian Clinical Toxicology Research Collaboration, and Burns, Trauma and Critical Care Research Centre, University of Queensland, Brisbane, Australia; c South Asian Clinical Toxicology Research Collaboration, and Professorial Medical Unit, University of NSW, Sydney, Australia; d Scottish Poisons Information Bureau, Royal Infirmary, and Clinical Pharmacology Unit, University of Edinburgh, UK; e Bundeswehr Institute of Pharmacology and Toxicology, Munich, Germany; f Walther-Straub-Institute of Pharmacology and Toxicology, Ludwig Maximilians University, Goethestr. 33, D-80336 Munich, Germany

**Keywords:** AChE, acetylcholinesterase (EC 3.1.1.7), BChE, butyrylcholinesterase (EC 3.1.1.8), CPF, chlorpyrifos, CPO, chlorpyrifos oxon, PON 1, paraoxonase 1 (EC 3.1.8.1), RBC, red blood cells, Organophosphorus, Chlorpyrifos, Poisoning, Toxicokinetics, Pralidoxime

## Abstract

Chlorpyrifos (CPF) is a pesticide that causes tens of thousands of deaths per year worldwide. Chlorpyrifos oxon (CPO) is the active metabolite of CPF that inhibits acetylcholinesterase. However, this presumed metabolite has escaped detection in human samples by conventional methods (HPLC, GC-MS, LC-MS) until now. A recently developed enzyme-based assay allowed the determination of CPO in the nanomolar range and was successfully employed to detect this metabolite. CPO and CPF were analysed in consecutive plasma samples of 74 patients with intentional CPF poisoning. A wide concentration range of CPO and CPF was observed and the ratio of CPO/CPF varied considerably between individuals and over time. The ratio increased during the course of poisoning from a mean of 0.005 in the first few hours after ingestion up to an apparent steady-state mean of 0.03 between 30 and 72 h. There was a hundred-fold variation in the ratio between samples and the interquartile range (between individuals) indicated over half the samples had a 5-fold or greater variation from the mean. The ratio was independent of the CPF concentration and the pralidoxime regimen. CPO was present in sufficient quantities to explain any observed acetylcholinesterase inhibitory activity. The effectiveness of pralidoxime in reactivating the inhibited acetylcholinesterase is strongly dependent on the CPO concentration. Differences in clinical outcomes and the response to antidotes in patients with acute poisoning may occur due to inter-individual variability in metabolism.

## Graphical abstract

Formation of chlorpyrifos oxon (CPO) in self-inflicted poisoning with chlorpyrifos (CPF).

Left: time course of plasma concentrations (single patient). Right: ratio of CPO/CPF 2–5 days after poisoning (*n* = 74).

## 1 Introduction

Chlorpyrifos (CPF) is one of the most commonly used organophosphorus pesticides for domestic, agricultural and industrial purposes. It may be estimated that it causes tens of thousands of deaths per year worldwide [1,2]. CPF is a pro-poison that requires metabolic activation to become a potent phosphorylating agent, namely chlorpyrifos oxon (CPO) [3]. At low concentrations, the desulfuration of CPF by human liver microsomes is due to the action of the polymorphically expressed CYP2B6 owing to its favorable *V*_max_/*K*_m_ ratio [4]. At high poison concentration, however, an increasing proportion of CPF desulfuration occurs due to the abundant CYP3A4 [5]. CPO is also rapidly detoxified by human liver microsomes via CYP-dependent deethylation and dearylation, and by glutathione-S-transferase [6]. In addition, reactions with A-esterases such as paraoxonase 1 (PON 1; EC 3.1.8.1) or B-esterases such as carboxylesterase (EC 3.1.1.1) and butyrylcholinesterase (BChE; EC 3.1.1.8) in the liver may rapidly degrade or scavenge CPO [7]. These polymorphisms in the activating and detoxifying enzymes [8] are expected to lead to wide inter-individual variability in exposure to CPO for a given CPF load. However, further exploration of the importance of these factors has been limited by the absence of a reliable method of quantifying CPO *in vivo*.

Until recently [9], CPO had never been detected in the blood of CPF-poisoned patients [10,11]. Yet we repeatedly observed that plasma of CPF-poisoned patients was able to inhibit red blood cell (RBC) acetylcholinesterase (AChE; EC 3.1.1.7) from healthy donors *in vitro*. It had been noted that BChE had extremely high reaction rates with CPO [12] and an enzyme-based assay was developed using BChE (described in detail in [9]). In the current study, we determined the ratio of CPO/CPF in patients with moderate to severe self-inflicted poisoning.

## 2 Methods

### 2.1 Patients

Serial blood samples stem from patients with acute chlorpyrifos poisoning who were enrolled in two randomised controlled studies (RCT1: ISRCTN02920054 and RCT2: ISRCTN55264358). The studies were performed in Sri Lankan hospitals in Anuradhapura and Polonnaruwa aimed at evaluating the efficacy of oral superactivated charcoal (RCT 1) or pralidoxime (RCT 2) in symptomatic organophosphorus pesticide poisoning. Additional patients were recruited in a prospective cohort study in Nuwara Eliya that was assessing changes in neuromuscular junctional function in organophosphorus pesticide poisoning. Ethics approval was obtained from the Faculty of Medicine Ethics Committee of Universities of Colombo and Peradeniya, and Oxfordshire Clinical Research Ethics Committee. Written informed consent was taken from each patient, or their relatives, in their own language. Study details are reported elsewhere [2,13,14]. Treatment consisted of fluid resuscitation and atropine as described [15] and pralidoxime chloride 1 g every 6 for 48 h (RCT1 and Nuwara Eliya cohort), although there was no strict adherence to this regimen. Patients in RCT2 were randomised to receive pralidoxime (2 g pralidoxime chloride loading dose over 20 min, then 0.5 g/h until a maximum of 7 days) or an equal volume of normal saline in addition to the supportive therapy mentioned above. The blood samples were collected between 25 May 2004 and 18 October 2006.

Blood samples were collected into tubes containing EDTA prior to pralidoxime, then at 1, 4, 12, and 24 h after administration, then once daily until discharge or death. A 0.2 mL blood sample was diluted at the bedside immediately after collection with 4 mL of ice-cold saline and frozen to −23 °C until analysis of AChE activity. The remaining sample was promptly centrifuged and the aspirated plasma frozen at −23 °C until analysis of BChE activity, inhibitory activity, and the concentration of CPF and CPO [15].

### 2.2 Analyses

Laboratory analyses were conducted in Munich, Germany. AChE activity was determined in native RBCs (AChE *in vivo*) and after reactivation with supra-therapeutic obidoxime (AChE *in vitro*) to estimate the reactivatable fraction. These assays were performed using a modified Ellman assay as described [16]. BChE activity [15] and inhibitory activity of plasma [17] were assessed as reported. In short, inhibitory activity of patient plasma was tested by incubating 3 volumes of hemolysate (3 g Hb/100 mL 0.1 M phosphate buffer, pH 7.4; washed red blood cells from a healthy donor) with 1 volume of patient plasma at 37 °C for 60 min.

CPF was determined by reversed phase HPLC with detection at 288 nm. Specifically, 0.5 mL plasma was mixed with 10 μL of 500 μM fenitrothion as internal standard to control the extraction yield. The plasma was extracted twice with 1 mL n-hexane for 1 min and residual lipoproteins in the combined hexane phases were precipitated with 50 μL 1 M perchloric acid, followed by centrifugation (10.000 rpm for 1 min). The hexane phase was transferred in a borosilica vial and 10 μL of DMSO was added as keeper. Hexane was evaporated at approximately 40 °C for 30 min in a Speed-Vac-Concentrator. The residue was dissolved in 90 μL of methanol:water (70/30, v/v) and 50 μL were applied to LiChrosphere 100 RP-18, 5 μm, 125 mm × 4 mm (E. Merck, Darmstadt, Germany) and eluted at a flow rate of 1 mL/min by gradient elution. The mobile phase started with 65% methanol in water and was linearly increased to 77% methanol between 4.5 and 7.5 min. Typically, fenitrothion was eluted after 3.3 min and chlorpyrifos after 11.5 min. The lower limit of quantification (LOQ) was 0.1 μM in plasma.

CPO was determined by an enzyme-based assay as described recently [9]. In short, CPO was extracted with n-pentane and the reconstituted residue titrated with purified horse BChE, allowing an LOQ of 0.5 nM. The data were corrected for 83% recovery of CPO from plasma [9].

Horse BChE (Cat. No C-1057) and CPF Pestanal^®^ were from Riedel-de Haen, Sigma, Taufkirchen, Germany; CPO was a generous gift from DowAgroSciences, Indianapolis, IN. Both compounds had purities ≥99%. Fenitrothion (95.5% pure) was obtained from Dr. Ehrenstorfer, Augsburg, Germany. All other reagents were from commercial sources at the purest grade available.

Pralidoxime was determined by HPLC on reversed phase material with an ion-pairing reagent. Specifically, 100 μL plasma was mixed with 30 μL trichloroacetic acid (1 M) and spun down. Eighty microliter of supernatant was mixed with 40 μL of n-heptanesulfonate reagent (PIC B7 low UV, Millipore) and 30 μL of a sodium formate buffer (1 M, pH 3.5) to give a final pH ≥ 3.0. Fifty microliter was applied to a Purosphere Star RP-18 column (55 mm × 4 mm, 3 μm, Merck) and eluted with a linear methanol gradient (0–15% within 7.5 min) in 5 mM PIC-B7 low UV reagent in water at a flow rate of 1.4 mL/min (20–25 °C) and detection at 293 nm. Typically, pralidoxime was eluted after 5.6 min. LOQ was 1 μM pralidoxime in plasma.

Free EDTA in plasma samples was titrated with a standard calcium chloride solution (50 mM) in the presence of the calcium indicator eriochrome black T (CAS 1787-61-7; 10 mg eriochrome black + 1 g sodium chloride triturated and dissolved in 2.5 mL water). Specifically, 50 μL plasma was diluted with 450 μL distilled water, 5 μL sodium hydroxide (2 M) and 1 μL indicator solution added and titrated with calcium chloride until formation of a pink coloration.

### 2.3 Calculations

Data were included when CPF was ingested (on the basis of history and/or analytical data) with no other organophosphorus compound being detected, BChE was mostly inhibited (typical for CPO) and AChE not significantly aged (typical for a diethylphosphorylated enzyme). Data were excluded when CPF and/or CPO were below the LOQs or when the time of ingestion or blood sampling was ambiguous. Data were also excluded when plasma pralidoxime was higher than 0.5 mM, indicating that blood was withdrawn proximally from the infusion site. In these cases significant post-sampling reactions had to be suspected.

The relationship between measured and calculated (predicted) CPO concentrations was determined using equations as deduced elsewhere [18]. This approach has been validated for paraoxon previously [19]. The basis for the calculation of free CPO is the equation given below:

$$\frac{\left[\text{E}\right]}{\left[\text{EP}+\text{EPOx}\right]}=\frac{k_{\text{r}}}{k_{\text{i}}\times [P]\times (1+K_{\text{D}}/[\text{Ox}])}$$

E = active enzyme; EP = phosphorylated, but unaged enzyme; EPOx = complex of oxime and phosphorylated, but unaged enzyme; [EP + EPOx] is the reactivatable enzyme = AChE *in vitro*–AChE *in vivo*; *k*_r_ is the first-order reactivation rate constant; *K*_D_ is the dissociation constant of the complex EPOx; *k*_i_ is the second-order inhibition rate constant; [P] is the oxon concentration; [Ox] is the oxime concentration.

The constants for reactivation of human diethylphosphoryl-AChE by pralidoxime are known from *in vitro* experiments using human red blood cells [18,19], [E], [EP + EPOx] and [Ox] were determined, hence [P] can be calculated. We used *k*_r_ = 0.3 min^−1^; *K*_D_ = 330 μM and *k*_i_ = 6 × 10^6^ M^−1^ min^−1^ [18].

The fraction of active test AChE upon incubation with known amounts of CPO in plasma samples was calculated discretely in 1-min increments using the following equation:

$$E=E_{0}-k_{\text{i}}\times (E-\text{EP})\times 0.15\times (\text{CP}{\text{O}}_{0}-\text{EP})\text{,}$$

with *E*_0_ and CPO_0_ being the AChE and total CPO concentration at *t* = 0 (the factor 0.15 corrects for the fraction not bound reversibly to albumin).

Calculations were done with Microsoft Excel 2004. Correlation analyses, Mann–Whitney U test and graphical presentations were performed with Prism4, GraphPad, San Diego, CA.

## 3 Results

### 3.1 Time course of CPO formation

**Fig. 1:**
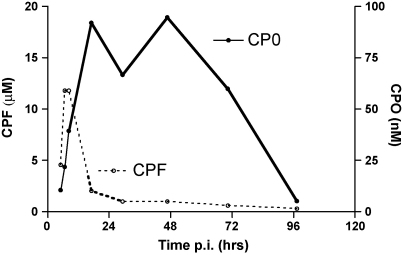
Time course of CPF and CPO concentrations in a patient with CPF self-poisoning. The patient did not receive pralidoxime. *X*-axis shows the time post-ingestion. Note the different concentration scales.

**Fig. 2:**
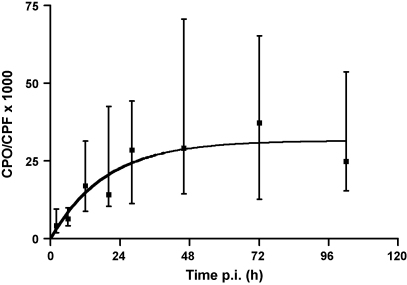
Ratio of CPO/CPF vs. time post-ingestion of CPF. Data are median values with their IQRs of 72 eligible patients from Table 1. For illustration a mono-exponential association function was fitted to the median values.

Formation of CPO occurred quite early after CPF ingestion. In one patient we detected significant amounts of CPO 70 min after CPF ingestion with BChE and AChE depressed to less than 2% and 10% of normal, respectively. Fig. 1 shows the time course of plasma CPF and CPO in a patient who was admitted some 5 h post-ingestion and received no pralidoxime. Here, maximal CPO concentration appeared after a delay of several hours. As expected, the ratio of CPO/CPF increased with time, starting with less than 0.005 in the period between 0 and 4 h after ingestion and approaching 0.03 on average in the period between 24 and 120 h. Table 1 shows the median plasma concentrations along with the interquartile ranges of CPF and CPO in 72 eligible patients (with and without pralidoxime treatment, no significant difference), where sufficient data were available to determine the CPO/CPF ratio at various time intervals post-ingestion. Fig. 2 shows the time course of the ratio of CPO/CPF and a mono-exponential association curve that was fitted to the median values, without implying a specific mechanism.

**Fig. 3:**
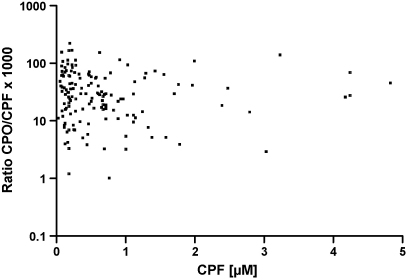
Ratio of CPO/CPF vs. CPF concentration in patient samples 24–120 h post-ingestion from the dataset shown in Fig. 2. Linear regression analysis of the logarithmically transformed ratio vs. CPF concentration gave a slope not significantly different from zero.

The variation of the ratio CPO/CPF was large, and some individuals showed a ratio below 0.01, while others had >0.05 in the period between 24 and 120 h, when a steady-state was reached (cf. Fig. 2). The ratio CPO/CPF showed no dependence on the CPF concentration. When plotting the ratio CPO/CPF in a logarithmic scale vs. the CPF concentration (Fig. 3), the linear regression showed no significant deviation of the slope from zero (*p* > 0.4; not shown). These data were indicative that in this setting CPO formation and decomposition did not show a saturation (dose-limiting) phenomenon.

### 3.2 Dependence of AChE-inhibitory material in plasma on CPO concentration

**Fig. 4:**
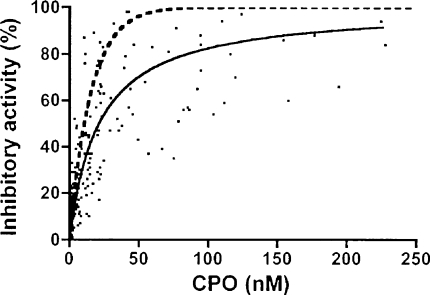
Inhibitory activity of patient's plasma towards RBC–AChE and its dependence on the measured concentration of CPO. Plasma samples (*n* = 241 of 67 patients), where pralidoxime was negative, were incubated with 3 volumes of haemolysate of an unexposed donor for 60 min and the degree of AChE inhibition determined. For illustration a rectangular hyperbola was fitted to the data points (solid line). Using the inhibition rate constant of human red blood cell AChE allowed the calculation of the expected curve (dotted line).

**Fig. 5:**
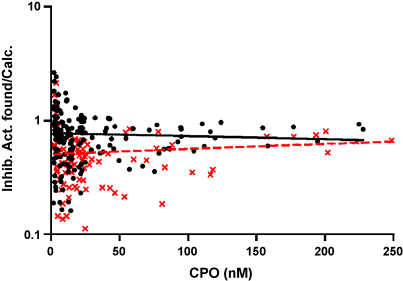
Semilogarithmic plot of the ratio of found and expected inhibitory activity of plasma from CPF-poisoned patients vs. the total CPO concentration. Closed circles and full line from pralidoxime-free samples; Xes and broken line samples containing pralidoxime between 13 and 150 μM.

A large scattering of data was observed in the ratio of AChE-inhibitory activity of patients’ plasma and the CPO concentration, whether or not patients had received pralidoxime. As expected, the inhibitory activity was on average higher in the group who did not receive pralidoxime. Fig. 4 shows the scatter of data from patients who were not administered pralidoxime (absence confirmed by HPLC). A hyperbola fitted by non-linear regression is shown (full line). The normal concentration of AChE in whole blood is about 15 nM [20,21], hence the concentration of AChE in the inhibition assay was about 11.3 nM. Using the inhibition rate constant of 6 × 10^6^ M^−1^ min^−1^ [18] allows a rough estimate of the inhibition progress, which was essentially complete after the 60 min incubation time. As shown in Fig. 4, in most cases AChE was inhibited to a less extent than predicted from the CPO concentration (dotted line). The ratio of inhibitory activity found/predicted in samples with and without PAM did not depend on the CPO concentration as shown in the semilogarithmic plot of Fig. 5. Linear regression analysis of the data indicated that the slopes were not significantly different from zero (*p* >0.2 and >0.5, respectively). These results pointed to competing side reactions of CPO during the incubation period.

Since BChE was essentially completely inhibited at even low concentrations of CPO we suspected that OP-hydrolases were degrading CPO in the inhibition assay. PON 1 is notorious for its high activity towards CPO [22]. This enzyme, however, requires free calcium ions, both for stability and activity [23]. Hence, this enzyme should be inactivated in EDTA-based plasma samples. We measured the free concentration of EDTA and found in 487 out of 490 plasma samples that excess EDTA was higher than 0.5 mM (median 4 mM). This should be sufficient to completely inactivate PON 1 and this implies other mechanisms of CPO inactivation/sequestration may exist.

### 3.3 Influence of CPO and pralidoxime concentrations on AChE activity

**Fig. 6:**
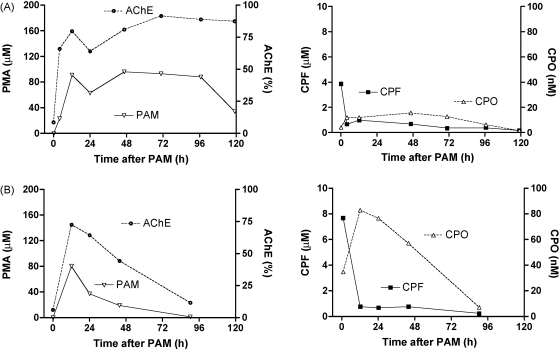
Influence of CPO and pralidoxime concentrations on AChE activity. Two patients, A and B, with a similar time course of plasma CPF had very different CPO concentrations in their plasma. Patient A received the intensified pralidoxime regimen with continuous infusion for >4 days; patient B received the intermittent bolus doses for <2 days, resulting in the re-inhibition of reactivated AChE. % AChE refers to the fraction of active enzyme from the pool of reactivatable AChE, thus ignoring the aged fraction.

Fig. 6 shows the effects of CPO and pralidoxime plasma concentrations on the percentage of active AChE in two selected patients with a similar time course of the CPF concentrations.

Patient A received pralidoxime by the continuous infusion regimen while patient B received the intermittent lower dose scheme. In both cases, AChE activity was largely inhibited before pralidoxime. In case A, AChE reactivation was sustained and approached some 90% when pralidoxime was given for 5 days. In contrast, intermittent doses of pralidoxime for less than 48 h resulted in gradual re-inhibition of reactivated AChE, particularly since CPO concentrations were much higher than in patient A.

**Fig. 7:**
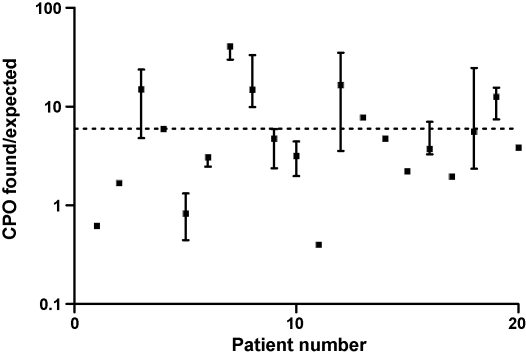
Ratio of found and expected CPO concentrations in 20 patients who were treated with pralidoxime. CPO was calculated from the ratio of active and inhibited, but reactivatable AChE, the measured pralidoxime concentration and the known kinetic constants as indicated in the Section 2. Data are given as median values (*n* ≥ 3; IQR) of 20 eligible patients during the expected steady-state of AChE inhibition and reactivation. Note the semilogarithmic scale. The dotted line intersecting the *Y*-axis at 6 corresponds to 85% reversible albumin binding of CPO in plasma, which is pharmacodynamically inactive, but was determined by solvent extraction.

The mutual dependence of the RBC–AChE activity on the pralidoxime and the CPO concentration was analysed according to the equation given in the Section 2. Forty-four patients who received pralidoxime, were eligible for the analysis contrasting the CPO concentration predicted from the RBC–AChE activity with the measured CPO. We excluded all data where CPO was below 0.5 nM (LOQ), pralidoxime given less than 1 h before sampling and where the pralidoxime concentration was below 13 μM. At this concentration the half-life for reactivation is approximately 1 h [19] and steady-state conditions could not be expected at lower concentrations. The expected free CPO concentration could be calculated from the ratio of inhibited, but reactivatable enzyme and the active enzyme, i.e. [EP + EPOx]/[E]. In doing so, we observed a large scattering of the ratio of CPO found/CPO predicted. Fig. 7 shows a plot of data (from 20 subjects with at least 3 samples that met the above criteria). The ratio of CPO found vs. CPO predicted is given in a logarithmic scale (median, IQR). The predicted CPO concentration is the free (active) fraction. Thus we expected a mean ratio of about 6 (dashed line), corresponding to 85% reversible albumin binding [7] as CPO from plasma was extracted by an organic solvent determining both the bound and free fractions of CPO.

## 4 Discussion

Since CPO reacts more than 2 orders of magnitude faster with BChE than with AChE [12], CPF-poisoned patients usually arrive at hospital with completely inhibited BChE while RBC–AChE is often less affected. These sequestering reactions along with the effective hydrolysis brought about by PON 1 were thought to keep the CPO steady-state concentration in blood too low for conventional determination (HPLC, GC-MS, LC-MS) [7,11]. However, in previous studies in CPF-poisoned patients [2] we observed that the patient's plasma was able to inhibit AChE of added test erythrocytes. The consistent presence of “inhibitory activity” of plasma led us to develop the enzyme-based assay for CPO.

Using the standard assay for CPO determination [9], reliable results were obtained in the concentration range of 2–20 nM CPO in plasma (accuracy ± 10%, reproducibility ± 4% (95% CI)). When it turned out that BChE was almost completely inhibited, plasma was diluted 1:10 or more with buffer to arrive at the useful range. Of course, the biological assay lacks specificity since all highly reactive inhibitors that are extracted with n-pentane may mimic CPO. With the exception of the nerve agents soman and cyclosarin no oxons are so reactive as CPO in inhibiting BChE [12,24,25]. Of the insecticidal organophosphates diazoxon is next reactive, but still 20-times less than CPO [24]. We intentionally minimized the reaction time for BChE inhibition to largely exclude the effect of potential cross-reacting oxons. Significant CPF concentrations were verified in all patients included and the presence of the lipophilic organophosphates fenthion and quinalphos was excluded by HPLC analysis. Next, the presence of major concentrations of dimethylphosphoryl compounds could be ruled out because erythrocyte AChE did not indicate the rapid ageing seen with these compounds [15]. Earlier studies in Sri Lanka have shown that the alleged poison was confirmed in more than 90% of the patients [2]. Hence, we are confident that our assay detected in fact CPO and no other BChE inhibitors.

The present study confirms that CPO is present in more than sufficient quantities to be responsible for all the “inhibitory activity” of the plasma of CPF-poisoned patients. It also casts doubt on the accuracy of non-specific bioassays such as the *ex vivo* “inhibitory activity” and suggests that they may significantly and variably under-estimate the true extent of *in vivo* AChE inhibitory activity. As shown in Fig. 4 the correlation of CPO with inhibition of test AChE was not as strong as anticipated. Even if we assume that some 85% of CPO is bound to albumin [9], we would expect somewhat slower but finally complete inhibition of AChE if the molar concentration of CPO exceeded that of AChE (11.3 nM). This expectation was usually not met (Figs. 4 and 5), so it appears likely that CPO was subject to competing reactions *ex vivo*. Hydrolysis of CPO by PON 1 in the inhibition assay can be ruled out since EDTA in the plasma samples leads to complete inactivation of PON 1 [26]. Albumin, however, not only binds CPO reversibly, but also hydrolyses CPO as demonstrated by the liberation of 3,5,6-trichloropyridinol in the absence of free calcium ions [27]. This reflects the covalent binding of CPO to tyrosine 411 in human albumin [28]. Based on the assumption of an albumin concentration in human plasma of about 4 g/100 mL, our inhibition assay contained 1% albumin. It is known that bovine serum albumin ‘hydrolyses’ CPO at *V*_max_ of 0.9 nmol/min/mg protein with a *K*_m_ value of 0.4 mM at pH 7.4 and 37 °C [27]. Given that human serum albumin has similar kinetic constants, we can deduce that 20 nM CPO will be inactivated at a rate of 0.45 nM/min. This may be compared with the more rapid inhibition rate of 1.35 nM/min (11.3 nM AChE, 20 nM CPO, *k*_i_ = 6 × 10^6^ M^−1^ min^−1^, see above). It is clear that the (initial) inhibition rate will be slowed down upon gradual inhibition of AChE, while covalent binding to albumin that has a large molar excess of about 150 μM still proceeds. This latter pseudo-first-order reaction explains the observation that the consumption of CPO was largely independent of the CPO concentration (Fig. 5). If other plasma proteins were also involved in CPO binding the effect of competing reactions might be even greater. In fact, data exist showing that mouse plasma contains at least 11 proteins capable of binding organophosphorus compounds under physiological conditions [29].

The influence of pralidoxime on the inhibitory activity of the patient's plasma (Fig. 5) was only evident at low CPO concentrations, because the bioreactor AChE + pralidoxime has only a limited capacity to degrade CPO, due to the low molar AChE concentration present. The large scattering of the data at low-to-intermediate CPO concentrations (up to 100 nM CPO) in the presence of pralidoxime may be mainly caused by variations of its concentration in the plasma samples. Taken together in the absence of pralidoxime, roughly 2/3 of CPO in EDTA-based plasma was capable of inhibiting AChE, the remainder had most probably reacted covalently with other proteins with albumin being the most favorite candidate.

Appearance of CPO in plasma lags behind CPF. This may be due to delayed formation or, more probably, due to sequestering reactions with limited capacity. CPO is rapidly bound to B-esterases, such as BChE, AChE and carboxylesterase. The latter enzyme has a high binding capacity, which in the rat was estimated to be 3 orders in magnitude higher than that of BChE [11]. Circulating carboxylesterase is not found in human plasma [30], but located in tissues. When these B-esterases are saturated, free CPO may escape the major site of production, the liver. Concomitantly, CPO is prone to degradation by A-esterases (true hydrolysis) and by CYP450-mediated inactivation [11]. PON 1 has favorable kinetic properties towards CPO hydrolysis, but shows a polymorphism that affects differently the catalytic efficiency of organophosphates. Thus, PON 1 _192R_ is superior in hydrolyzing CPO and provides better protection against CPO exposure in *PON 1 null* mice than PON 1 _192Q_ [31]. Interethnic variability of the genetic makeup is remarkable in that *PON 1 RR* was rarely found in Indians (3%) but often in Chinese (32%), while the inverse relationship was found with the *PON 1 QQ* genotype [32]. In addition, a large variation in enzyme levels is found among individuals even with the same genotype [22]. Hence, steady-state concentrations of CPO (at constant CPF) are expected to vary widely. We observed steady-state ratios of CPO/CPF after some 30 h post-ingestion with a spread of the interquartile range of about 5 (cf. Fig. 2). The ratio was not dependent on the CPF concentration, indicating that the equilibrium of CPO formation and degradation did not show saturation phenomena. Desulfuration of CPF by human hepatic CYPs is mainly brought about by CYP2B6 and CYP3A4 with *K*_m_ values of about 1 and 30 μM, respectively, at comparable *V*_max_ values. Since the former CYP is prone to polymorphism in protein expression [33,34], large variability in oxon formation can be expected [4]. In fact, a variability of CPO/CPF of more than 1 order in magnitude was observed (Fig. 5).

Pralidoxime was able to reactivate inhibited RBC–AChE in CPF-poisoned patients as shown in Fig. 6. Its effectiveness depended both on its plasma concentration and on the CPO concentration. The two examples shown indicate that the variability in CPO production and also that if pralidoxime is ceased while there is measureable CPO present then the RBC–AChE will not remain reactivated. Such considerations are likely to apply to all OP, particularly when highly lipophilic agents are involved [2,15]. As deduced recently [18], the theoretical plasma concentration of CPO should be able to be calculated from the ratio of inhibited vs. active AChE, the plasma concentration of the oxime and the kinetic constants of the reactions involved (see Section 2). However, we observed a very large variation in the found/predicted CPO concentration ratio. We expected a mean ratio of about 6 (dashed line), corresponding to 85% reversible albumin binding [9] as CPO from plasma was extracted by an organic solvent determining both the bound and free fractions of CPO and the prediction only estimates free CPO. This variation seems much greater than variability in protein binding can explain but further studies are required to determine why the clinical samples show this discrepancy.

In conclusion, this newly developed CPO assay gives reliable results that enable a better assessment of the mechanisms behind inter-individual variation in susceptibility in CPF poisoning. The study has shown that the steady-state ratios of CPO/CPF vary widely and probably mirror the activating and detoxifying capabilities. Thus, a large variation in the susceptibility for CPF- and CPO-mediated toxicities has to be taken into account when debating safety margins of CPF.

## Figures and Tables

**Table 1:** Plasma concentrations of CPF and CPO after various time intervals post poisoning.

Time interval (h)	CPF (μM)	CPO (nM)	CPO/CPF × 1000	*n*
0–4	2.54 (1.6–4.7)	10.1 (4.5–22.2)	4.2 (1.9–9.5)	52
4–8	1.65 (0.7–3.2)	11.9 (6.5–21.5)	6.8 (4.3–14.0)	67
8–16	0.70 (0.4–1.2)	12.7 (5.5–22.5)	17.5 (9.2–31.6)	59
16–24	0.44 (0.3–1.5)	9.6 (4.1–30.1)	14.2 (10.5–42.6)	30
24–36	0.47 (0.3–1.1)	14.1 (4.7–34.5)	28.6 (11.3–44.4)	45
36–60	0.66 (0.2–1.0)	16.7 (3.4–68.4)	29.2 (14.4–70.7)	41
60–84	0.46 (0.2–0.8)	22.7 (2.2–59.9)	37.3 (12.7–65.3)	27
84–120	0.39 (0.2–0.7)	7.1 (4.2–40.2)	25.0 (15.4–53.6)	28

Plasma samples (*n* = 357) of 72 patients were eligible (CPF and CPO > LOQ). The ratio CPO/CPF was calculated from the individual sample values. Data are median values along with the interquartile range in brackets.
